# Possible Heterozygous Hypercholesterolemia Among Adults in Basrah, Southern Iraq

**DOI:** 10.7759/cureus.67625

**Published:** 2024-08-23

**Authors:** Elaf A Khamees, Nassar T Alibrahim, Abbas A Mansour

**Affiliations:** 1 Pathology, Faiha Specialized Diabetes, Endocrine and Metabolism Center (FDEMC), University of Basrah, Basrah, IRQ; 2 Diabetes and Endocrinology, Faiha Specialized Diabetes, Endocrine and Metabolism Center (FDEMC), University of Basrah, Basrah, IRQ

**Keywords:** iraq, hypercholesterolemia, heterozygous, lipid, ldl-c, familial

## Abstract

Background

Familial hypercholesterolemia (FH) is an autosomal dominant disease and is one of the most prevalent genetic disorders. We aimed to determine the prevalence of patients with elevated levels of low-density lipoprotein cholesterol (LDL-C), presumptively indicating possible heterozygous familial hypercholesterolemia (HeFH).

Methods

Retrospective data analysis was conducted for adult patients aged 18 and above with fasting LDL-C ≥ 190 mg/dL registered in Faiha Specialized Diabetes, Endocrine and Metabolism Center (FDEMC) in Basrah for the period from August 2008 to December 2023. The total number of enrolled individuals was 59,026.

Results

From the data records of the 59,026 individuals enrolled in the study, it was found that 4,093 (6.9 %) had LDL-C levels ≥190 mg/dL and 361 (0.6 %) had very high total cholesterol according to the Make Early Diagnosis to Prevent Early Death (MEDPED) Criteria. Around 30,527 (51.7 %) were aged 40-59 years, representing the peak age group. Women comprised 34,688 (58.8 %), and 42,310 (71.7 %) had diabetes mellitus. Individuals with obesity comprise 27,375 (48.8 %). Out of the 4,093 patients with LDL-C ≥190 mg/dL, 2,422 (59.2 %) were in the 40-59 years age group, and 2,847 (69.6 %) were women. Diabetes was found in 3,442 (84.1 %) patients and obesity in 1,954 (49.9 %) patients. The average blood pressure was higher in the individuals with LDL-C ≥190 (137/83 versus 134/82 respectively, p < 0.001).

Conclusions

Being one of the largest studies of its kind in the country, the percentage of individuals who might meet the criteria for having possible HeFH in Basrah (Southern Iraq) should raise awareness about the size of the problem in the country, both to encourage family screening programs and to broaden the need for lipid-lowering therapies. Future studies are needed to have further clarifications about the differences in the prevalence between sexes and age groups. These findings need further confirmation by genetic studies including LDL-receptor mutations.

## Introduction

Familial hypercholesterolemia (FH) is an autosomal dominant disease and is one of the most prevalent genetic disorders characterized by markedly elevated levels of total cholesterol (TC) and low-density lipoprotein cholesterol (LDL-C) [1]. Heterozygous familial hypercholesterolemia (HeFH) affects approximately one in 200 to 500 people in the global population, equating to nearly 14 to 34 million individuals worldwide. HeFH is a highly common yet underdiagnosed disease [2].

The main criteria to define HeFH were the Simon Broome Criteria for the Diagnosis of HeFH (UK FH Registers Criteria) in 1991 [3], Make Early Diagnosis to Prevent Early Death (MEDPED) in 1993 [4], Dutch Lipid Clinic Network (DLCN) for Diagnosis of FH in 1998 [5], and American Heart Association in 2015 [6].

Definite HeFH criteria (according to the Simon Broome Familial Hypercholesterolemia diagnostic criteria) in adults include an LDL-C level ≥ 190 mg/dL plus at least one of two physical findings: tendon xanthomas or tendon xanthomas in first or second-degree relatives, or the presence of DNA-based evidence of an LDL-receptor mutation, familial defective apo B-100, or a PCSK9 mutation [7].

Possible HeFH in adults is indicated by LDL-C levels ≥ 190 mg/dL accompanied by at least one of the following criteria: family history of myocardial infarction at age 60 years or younger in a first-degree relative or at age 50 years or younger in a second-degree relative; family history of elevated TC exceeding 290 mg/dL in an adult first- or second-degree relative, or greater than 260 mg/dL in a child, sibling aged younger than 16 years, or brother/sister [7].

Ensuring the exclusion of potential secondary causes of severe hypercholesterolemia, such as hypothyroidism, nephrotic syndrome, and cholestatic hepatic disease, is essential prior to contemplating the diagnosis of HeFH [8]. The objective of this study was to determine the prevalence of patients with LDL-C levels ≥ 190 mg/dL, presumptively indicating possible HeFH, as per the Dutch Lipid Clinic Network (DLCN) criteria for diagnosing HeFH [5].

## Materials and methods

Design and settings

Retrospective data analysis was conducted for patients with fasting LDL-C ≥ 190 mg/dL registered in Faiha Specialized Diabetes, Endocrine and Metabolism Center (FDEMC) in Basrah (Southern Iraq) for the period from August 2008 to December 2023. FDEMC is a tertiary referral center [9].

Data collection

Data were extracted from the FDEMC database; for all registered individuals who had a lipid profile done for any reason including screening, we included the records of age, sex, smoking status, clinical notes, diagnosis, BMI, blood pressure, and lipid profile.

Inclusion criteria

Adult patients aged 18 and above presenting with a minimum of eight hours of fasting who had done a lipid profile for any reason found in the registry of FDEMC from August 2008 to December 2023 were included.

Exclusion criteria

**Figure 1:**
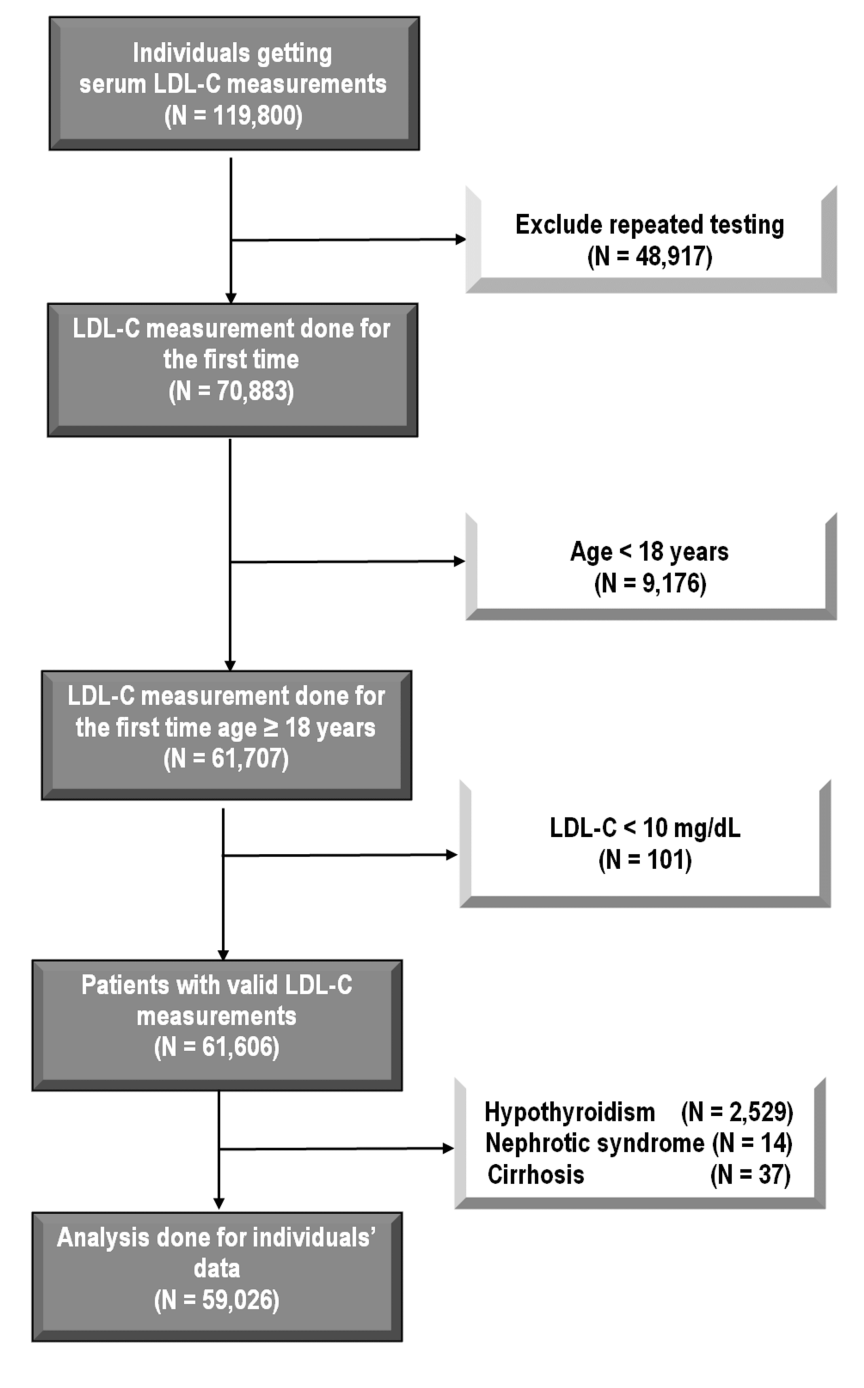
Study flow chart LDL-c: Low-density lipoprotein cholesterol

We excluded any repeated lipid profile measurements (those readings in the follow-up visits) and we depended only on the first readings. Reading with invalid LDL-C levels (like LDL-C < 10 mg/dL) were excluded as well. Besides, 2529 were excluded because of hypothyroidism, 14 because of nephrotic syndrome, and 37 because of liver cirrhosis (Figure 1).

Definition of possible HeFH was based on the Dutch Lipid Network Criteria [5] for diagnosis of HeFH because the data we collected were retrospective and no points in the history were taken about tendon xanthomas, corneal arcus occurring in individuals younger than 45 years old, and premature cardiovascular disease (CVD) in the patients or his relatives. The identification of mutations in the LDL-R, ApoB, or PCSK9 gene was not possible nationwide.

Diabetes mellitus (DM) definition was based on the American diabetes association criteria for new cases or diagnosed cases and treatment including medical nutritional therapy alone. Hypertension was defined as systolic blood pressure (SBP) in the ofﬁce of ≥140 mm Hg and or diastolic BP (DBP) ≥90 mm Hg or on medical treatment. Ischemic heart disease was defined as either a self-reported diagnosis by a physician of myocardial infarction, angina, or heart failure, and/or documentation of any of these conditions in the patient's medical records. Cerebrovascular disease was determined based on the presence of sudden neurological symptoms and signs, which may or may not have been confirmed by neuroimaging. Body mass index (BMI) was calculated as weight (in kilograms) divided by squared height (in meters squared).

The study was done in accordance with the ethical standards of Faiha Specialized Diabetes, Endocrine and Metabolism Center (FDEMC) Research and Ethical Committee, from which the ethical approval was obtained under the reference (24/15/53).

Biochemical analysis

The lipid profile was measured in all individuals in a fasting state (minimum eight-hour fast). In the morning, 5 mL of venous blood was taken in a gel-activated tube, and the assay was done using a Roche Cobas C311 chemistry analyzer (Roche Diagnostics, Switzerland). A lipid profile assay was done for TC, estimated LDL-C, triglyceride (TG), and high-density lipoprotein cholesterol (HDL-C). Non-HDL-C was determined from the lipid profile using the formula: non-HDL-C = total cholesterol minus HDL-C, and this was the consistent laboratory practice of our center for many years.

Statistical analysis

All data was entered and processed using IBM SPSS Statistics for Windows, Version 25 (Released 2017; IBM Corp., Armonk, New York, United States) for analysis. Continuous variables were summarized as mean± SD, while categorical variables were presented as percentages. Group differences were assessed using the Student t-test for continuous data and either the X2 test or Fisher’s exact test (for small sample sizes) for categorical data. A P-value of < 0.05 was used as the statistical significance level.

## Results

About 4,093 (6.9%) out of 59,026 individuals studied had LDL-C levels ≥190 mg/dL (Table 1). Of the total enrolled, 28,105 (51.7 %) were aged 40-59 years, representing the peak age group. Women comprised 34,688 (58.8 %). There were 42,310 (71.7 %) patients with diabetes, with 1,742 / 42,310 (4.1 %) with type 1 diabetes and 40,568 / 42,310 (95.9 %) with type 2 DM. The mean SBP was 134.8 ± 20.7 mm Hg, and the mean DBP was 82.3 ± 12.1 mmHg. Hypertension was present in 26,756 individuals (45.3 %), while 1,203 (2.0 %) had cerebrovascular disease and 3,862 (6.5 %) had ischemic heart disease. Additionally, 6,564 (11.1 %) individuals were smokers. The mean BMI was 30.3 ± 6.6 kg/m² for the studied individuals. Individuals with LDL-C levels ≥190 mg/dL were more commonly aged 40-59 years, women, diabetic, and exhibited higher systolic and diastolic blood pressure. Furthermore, they were less likely to smoke. The BMI (kg/m^2^) was not different among groups.

**Table 1:** Comparison between patients with LDL-C <190 mg/dL and those with LDL-C ≥190 mg/dL among 59,026 individuals. LDL-C: Low-density lipoprotein cholesterol, DM: diabetes mellitus, BMI: body mass index. The mean ±SD of the blood pressure (systole/diastole in mm Hg) for all individuals was 134.8±20.7 / 82.3±12.1. For patients with LDL-C ≥190 mg/dL, it was 137.1±21.4 / 83.8±12.3 in comparison to 134.6±20.6 / 82.2±12.1 for patients with LDL-C <190 mg/dL (p-value <0.001*). The mean ±SD of the BMI (in kg/m^2^) for all individuals was 29.8±6.6. It was 30.5±6.1 for individuals with LDL-C ≥190 mg/dL and 30.3±6.6 for those with LDL-C < 190 mg/dL (p=0.171) Data are expressed as N(%). *significant p-value **Of all patients with DM.

Variables		Total N (%)	LDL-C (mg/dL)		p-value
			<190	≥190	
Total		59,026	54,933 (93.1)	4,093 (6.9)	<0.001*
Age (years)	18-39	15,780 (26.7)	15,129 (95.9)	651 (4.1)	
	40-59	30,527 (51.7)	28,105 (92.1)	2,422 (7.9)	
	60-79	12,357 (20.9)	11,361 (91.9)	996 (8.1)	
	≥80	362 (0.6)	338 (93.4)	24 (6.6)	
Gender	Men	24,338 (41.2)	23,092 (94.9)	1,246 (5.1)	<0.001*
	Women	34,688 (58.8)	31,841 (91.8)	2,847 (8.2)	
DM	Yes	42,310 (71.7)	38,868 (91.9)	3,442 (8.1)	<0.001*
	No	16,716 (28.3)	16,065 (96.1)	651 (3.9)	
	Type 1 DM	1,742 (4.1)**	1,624 (93.2)	118 (6.8)	0.034*
	Type 2 DM	40,568 (95.9)**	37,244 (91.8)	3,324 (8.2)	
Hypertension	Yes	26,756 (45.3)	24,595 (91.9)	2,161 (8.1)	<0.001*
	No	32,270 (54.7)	30,338 (94.0)	1,932 (6.0)	
Cerebrovascular disease	Yes	1,203 (2.0)	1,108 (92.1)	95 (7.9)	0.184
	No	57,823 (98)	53,825 (93.1)	3,998 (6.9)	
Ischemic heart disease	Yes	3,862 (6.5)	3,592 (93.0)	270 (7.0)	0.885
	No	55,164 (93.5)	51,341 (93.1)	3,823 (6.9)	
Smoking	Yes	6,564 (11.1)	6,211 (94.6)	353 (5.4)	<0.001*
	X-Smokers	3,025 (5.1)	2,845 (94.0)	180 (6.0)	
	No	49,437 (83.8)	45,877 (92.8)	3,560 (7.2)	
BMI (kg/m^2^)	<30	28,770 (48.7)	26,811 (93.2)	1,959 (6.8)	0.126
	>=30	27,375 (46.4)	25,421 (92.9)	1,954 (7.1)	

In Table 2, LDL-C levels in mg/dL were stratified into five categories. Among them, 45,606 (77.3 %) had LDL-C <155; 9,325 (15.8 %) had LDL-C between 155 and 189; 3,649 (6.2 %) had LDL-C between 190 and 249; 407 (0.7 %) had LDL-C between 250 and 329, and 39 (0.1 %) had LDL-C >330 mg/dL.

**Table 2:** Spectrum of LDL-C levels. LDL-C: Low-density lipoprotein cholesterol.

LDL-C levels (mg/dL)	Total N(%)
<155	45,606 (77.3)
155–189	9,325 (15.8)
190–249	3,649 (6.2)
250–329	407 (0.7)
>330	39 (0.1)

In Table 3, there were 22 individuals with age 18-19 years and TC ≥270 mg/dL, 74 individuals with age 20-29 years and TC ≥ 290 mg/dL, 47 individuals with age 30-39 years and TC ≥340 mg/dL, and 218 individuals with age ≥40 years and TC ≥360 mg/dL. This means that there were 361 individuals (0.6%) out of 59,026 which was approximately 1:164 with very high TC.

**Table 3:** Spectrum of TC levels in mg/dL according to the age group. TC: Total cholesterol.

Age (years)	TC (mg/dL)	Total N
18-19	≥270	22
20-29	≥ 290	74
30-39	≥340	47
≥40	≥360	218
Total N (%)		361/59,026 (0.6)

## Discussion

This was the first study of its type to start searching for the epidemiology of HeFH in Iraq. We found in this study that 4,093 of the 59,026 individuals studied had LDL-C levels ≥190 mg/dL. This could mean that 6.9% of the studied population had met the criteria of possible HeFH using the Dutch Lipid Clinic Network [5].

If we apply the US MEDPED criteria for FH Diagnosis [4], approximately 361 (0.6 %) out of 59,026 individuals might meet the criteria for HeFH, which roughly translates to one in every 164 individuals. So between 6.9 % and 0.6 %, we have to reassess the prevalence of HeFH in our country.

The multicenter, multinational Gulf FH registry found that HeFH prevalence was 0.9 % (1:112) among adults in Arabian countries in the Gulf region in the Middle East [10]. That study was over five years and included genetic confirmation. Two studies on HeFH from Egypt and Iran included history and clinical examination without genetic study reported [11,12]. Among 600 premarital couples in Qalubya Governorate in Egypt, 3 (0.5 %) had possible HeFH [11].

In Iran, among the 997 individuals included in a registry, a total of 263 patients were diagnosed with probable or deﬁnite HeFH, and others were in the possible group [12]. In a large Pakistani study by Farhad et al., which was laboratory-based and encompassed 988,306 unique individuals across Pakistan, a total of 24,273 individuals (2.46 % or 1 in 40) were found to have an LDL-C level of ≥190 mg/dL which is lower than our study [13]. The prevalence of familial hypercholesterolemia based on MEDPED criteria was 0.24 % (1 out of 409) in a Pakistani study [4,13]. In our study, LDL-C ≥190 mg/dL was more in women while in the Pakistani study, it was more in men. By all means, the LDL-C level of ≥190 mg/dL is an absolute indication for starting lipid-lowering therapies regardless of risk factors [14].

Our finding of the higher prevalence of patients with LDL-C levels ≥190 mg/dL in the middle age group was in concordance with the finding of Alhabib et al., where the mean age for the patients with possible FH was 48 years [10], and with the finding of Farhad et al. where they found that around 72% of the patients were at the age of 40 years and above; on the other hand, the percentage of women in our study was similar to the finding of Alhabib et al. where women represented 54%, while in the Iranian study, there was no difference in the prevalence of possible FH between both sexes [10,12,13].

Compared to polygenic hypercholesterolemia, monogenic FH patients are at significantly higher risk for premature coronary heart disease, despite both diseases having comparable LDL-C levels in our study since no genetic analysis is available; that’s why we cannot differentiate between monogenic HeFH from polygenic hypercholesterolemia [15]. This could explain LDL-C levels ≥190 in 6.9 % of this study cohort. The absence of statistically higher rates of cerebrovascular disease and ischemic heart disease among those with LDL-C levels ≥190 mg/dL may be attributed to underreporting in a retrospective study with incomplete data. Studies on FH in Iraq are still in their infancy and need a lot of effort [16]. This study represented early awareness to assess the prevalence of HeFH in Iraq, but in the future should include clinical criteria, lipid profile, and if possible genetic criteria.

The limitations of this study include its retrospective design with no full history or family history of premature atherosclerotic CVD. Clinical examination for tendon xanthomas, tendon xanthomas in first or second-degree relatives, or arcus cornealis prior to age 45 years was not available for most. DNA-based evidence of an LDL-receptor mutation was not present in all. 

## Conclusions

Of the total enrolled individuals in this study, the number of those who had met the criteria for having possible HeFH despite being comparable to the findings in other countries in the region gave us more insight into the approximate size of the problem in the country. Being the first study of its kind in Iraq, it should raise awareness about the prevalence of possible HeFH in our country, both to encourage the policies to have more steps in screening family members in suspected individuals and to broaden the need for providing lipid-lowering therapies to the populations according to the guidelines. Future studies are needed to have further clarifications about the differences in the prevalence of FH between sexes and different age groups. A larger prospective study is needed to see the epidemiology of definite HeFH among the Iraqi population including not only proper personal or family history and clinical examination but also the presence of functional LDL-R mutation.
